# Covid-19 and the Subsequent Lockdown Modified Dietary Habits of Almost Half the Population in an Italian Sample

**DOI:** 10.3390/foods9050675

**Published:** 2020-05-25

**Authors:** Federico Scarmozzino, Francesco Visioli

**Affiliations:** 1 Department of Molecular Medicine, University of Padova, Viale G. Colombo 3, 35121 Padova, Italy; federico.scarmozzino@studenti.unipd.it; 2 IMDEA-Food, CEI UAM + CSIC, 28049 Madrid, Spain

**Keywords:** Covid-19, diet, dietary habits, lockdown, food availability

## Abstract

The Covid-19 pandemic led to lockdowns in several parts of the world and, hence, changed some daily habits, including social interactions, the ability to perform sports, and—possibly—diet. The Italian government established and promulgated lockdown policies on 9 March 2020. We aim at assessing the effects of Covid-19-induced confinement policies on self-reported food consumption of self-selected Italians by means of a questionnaire that was created and diffused by the Internet. Nearly half, i.e., 49.6% of responders did not substantially modify their diet during the lockdown; however, 46.1% of them reported that they were eating more during confinement, and 19.5% gained weight. In particular, we report an increase in “comfort food” consumption, notably chocolate, ice-cream, and desserts (42.5%) and salty snacks (23.5%). In addition, 42.7% percent of this cohort attributed this increase to higher anxiety levels. Related to this, 36.8% of responders reported a decrease in alcohol consumption, even though 10.1% of them reported an increase. Interestingly, 21.2% of responders increased their consumption of fresh fruit and vegetables. Only 33.5% of those who declared decreased consumption attributed this change of diet to lower availability and ease of purchasing such items. Equally interesting, over half of responders, i.e., 56.2%, admitted that fruit and vegetables did not appeal to them while in lockdown. Purchases of ready-made meals were reduced by nearly 50%. Future large-scale similar studies should be undertaken worldwide and will help public health authorities shape their reactions to future, unavoidable pandemics.

## 1. Introduction

The Covid-19 pandemic led to lockdowns in several parts of the world and, hence, changed several daily habits, including social interactions, the ability to perform sports, and—possibly—diet. Diet being one of the foremost contributors to health [1], it is conceivable that a situation in which food availability, access to it, and a shift from eating out to mandatory in-house consumption could have change the dietary profiles of several people. However, to the best of our knowledge, this issue has never been explored.

One of the most interesting and novel proxies of healthy diets is food, namely, produce availability [2]. Interesting research is suggesting that the easier it is to purchase healthful foods, the easier it is to follow appropriate diets [3]. Food accessibility may be impaired during confinements, which could easily impact the overall diet quality. Moreover, the impending possibility of job losses, reduced incomes, and uncertainties regarding the future might speculatively lead some people to reduce their expenditures, including those for food.

Finally, lockdowns greatly reduce the amount of physical activity, sport, exercise, creating vicious cycles by which sub-optimal diets increase the noxious health effects of sedentarism [4]. This situation is exacerbated by severe restraints, such as those that are commonplace in prisons, where detainees often experience psychological distress [5]. Indeed, some authors [6] are proposing prospective neuropsychiatric monitoring of individuals exposed to SARS-CoV-2 at various points in the life course. Even though the neuropsychiatric burden of this pandemic is currently unknown, it is likely to be significant [6]. The relation between diet and mood or psychiatric status is mutual. On the one hand, some diets are being proposed as having positive effects on mood [7], possibly because they provide (poly)phenols (see below) [8], vitamins, and, e.g., tryptophan for serotonin production [9]. On the other hand, dietary choices are strongly influenced by psychological factors [10] in addition to the environment where we live or our beliefs [11]. In short, it is conceivable that lockdowns bring about dietary changes whose long-term health effects are unknown and worth investigating.

The Italian government established and promulgated lockdown policies on 9 March 2020. We aimed at assessing the effects of Covid-19-induced confinement policies on self-reported food consumption of self-selected Italians by means of a questionnaire that was created and diffused by the Internet.

## 2. Methods

On 3 April 2020, we created an anonymous questionnaire (in Italian, retrievable at https://clikka.net/0flBP) that aimed at assessing the most popular eating habits of Italians. Anonymity was guaranteed by the platform, and there was no way to link participants’ emails with responses. We distributed the questionnaire via social media, e.g., Linkedin, and published a link to it in a very popular Italian agriculture magazine (Olio Officina). Also, students from the Medical School of the University of Padova distributed the survey via personal contacts. Of course, participants were not rewarded, and we could not identify them, as mentioned above. We closed the survey and stopped collecting data on 15 April, when 1932 surveys were filled out. We then translated all questions and have provided them as Supplementary Materials.

We analyzed data immediately thereafter.

## 3. Results

**Figure 1:**
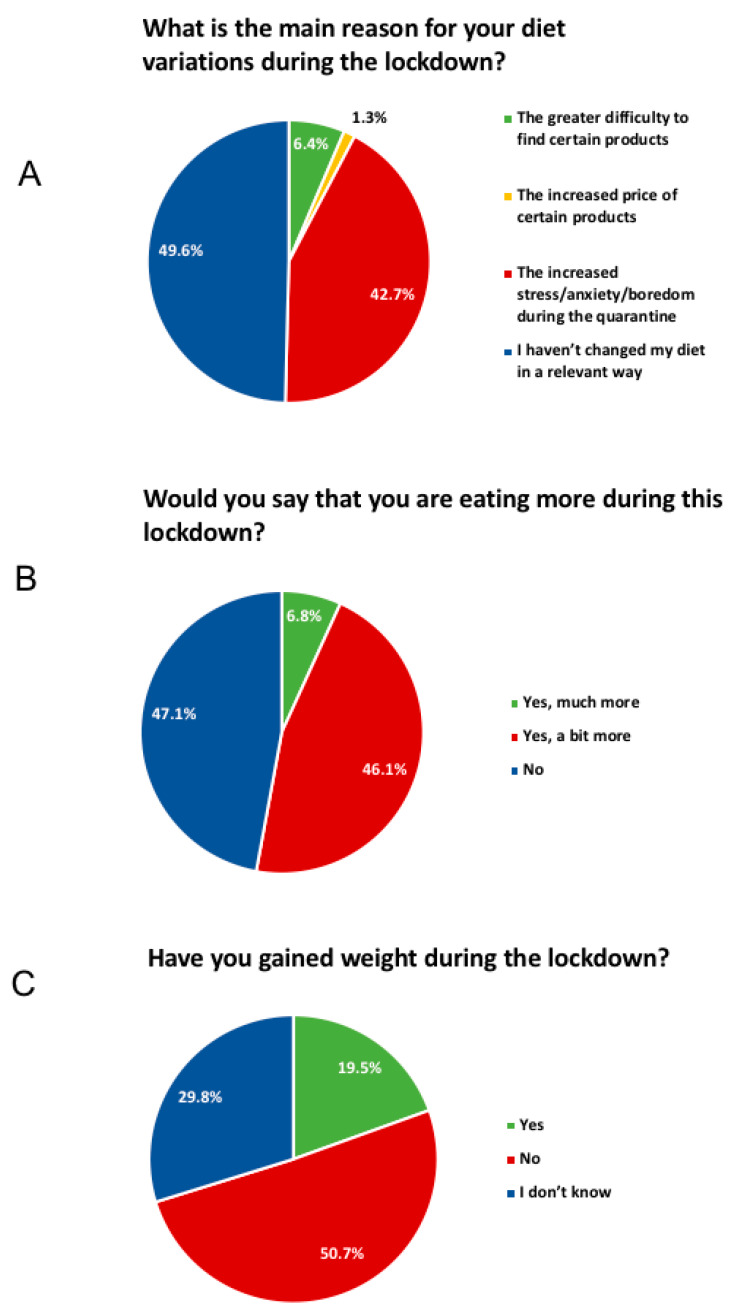
Overall reported dietary changes during the lockdown.

**Figure 2:**
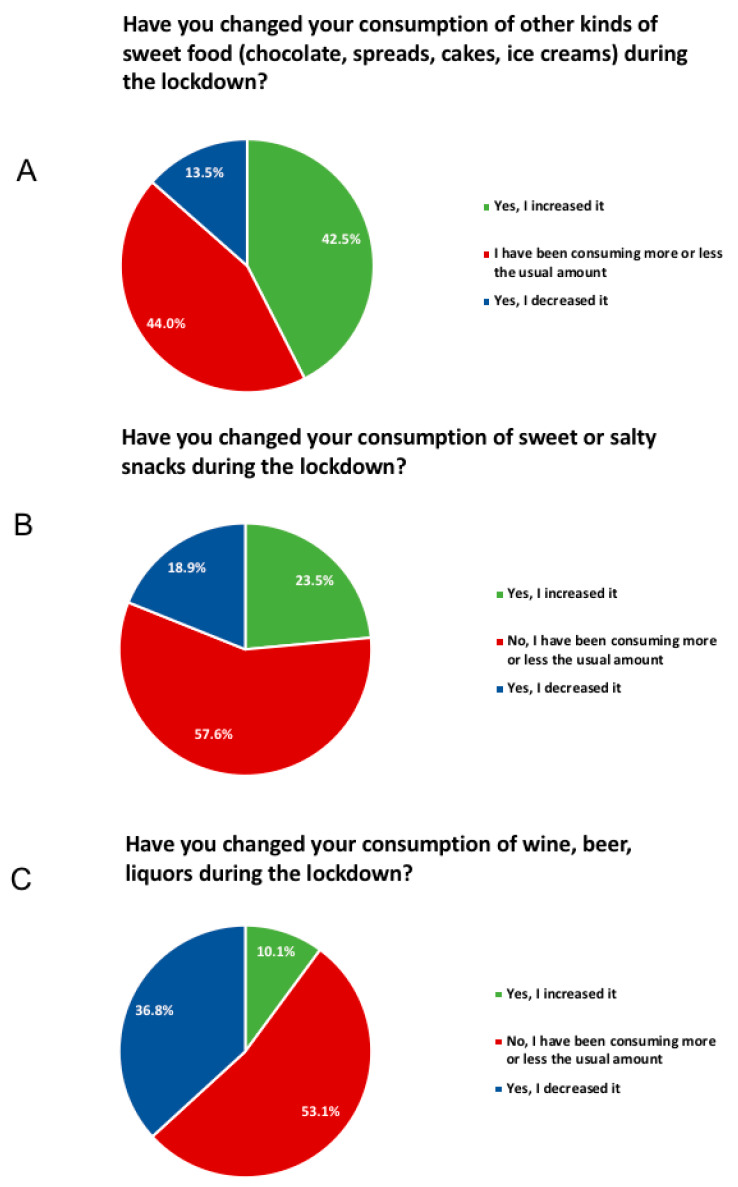
Reported changes in comfort food and alcohol consumption during the lockdown.

The entire dataset can be found in Figure S1. Here, we would like to focus on the most relevant results. Nearly half, i.e., 49.6%, of responders did not substantially modify their diet during the lockdown (Figure 1A); however, 52.9% of them reported that they were eating more during confinement (Figure 1B) and 19.5% gained weight (Figure 1C). In particular, we report an increase in “comfort food” consumption, notably chocolate, ice-cream, and desserts (42.5%) and salty snacks (23.5%) (Figure 2A,B). Furthermore, 42.7% percent of this cohort attributed this increase to higher anxiety levels. Related to this, 36.8% of responders reported a decrease in alcohol consumption (Figure 2C), even though 10.1% of them reported an increase.

**Figure 3:**
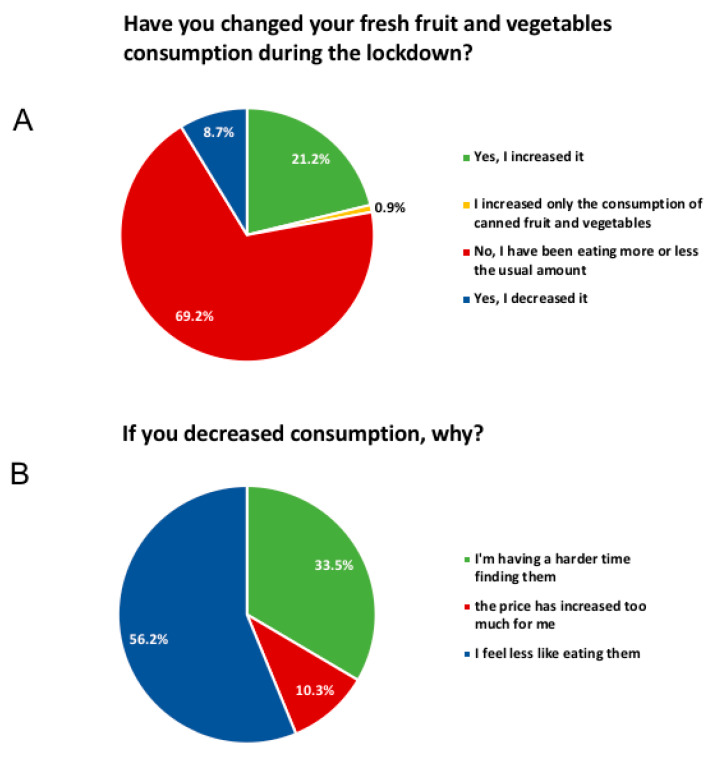
Reported produce consumption during the lockdown.

Interestingly, 21.2% of responders increased their consumption of fresh fruit and vegetables (Figure 3A). Only 33.5% of those who declared decreased consumption attributed this change of diet to lower availability and ease to purchase such items (Figure 3B). Equally interesting, over half of responders, i.e., 56.2%, admitted that fruit and vegetables did not appeal to them while in lockdown (Figure 3B).

Purchases of ready-made meals were reduced by nearly 50% (Figure S1).

## 4. Discussion

We surveyed the dietary habits of an Italian sample, via distribution of a questionnaire, during the Covid-19 pandemic. Our results indicate that Covid-19 and the subsequent lockdown induced about half the respondents to eat more. However, a relevant, i.e., 21.2% of responders upped their consumption of fresh fruit and vegetables. This is noteworthy in terms of micronutrient intake, which is particularly important in the elderly and in the population at large [12]. In terms of micronutrients, the lay public’s wisdom holds it that some vitamins, namely C and D, help fight viral infections. To date, however, the evidence is weak [13,14], and many more controlled trials should be performed. In any case, whether the protective effects of fruit and vegetables are due to their vitamins and other micro-nutrients [15] or fiber content or to their contribution to the improvement of the overall dietary pattern increased consumption should be viewed as positive.

In our sample, the majority, i.e., 64.8% of respondents did not remarkably change their habits of fish consumption (Figure S1), which was in line with the international guidelines for over 30% of the cohort (Figure S1) [16]. It is worth noting that the immunomodulatory effects of omega 3 fatty acids have never been explored in humans [17]. However, human immune cells are typically rich in arachidonic acid and poor in EPA and DHA [17]. In addition, the phospholipids of immune-competent cells (such as lymph node or splenic lymphocytes or peritoneal macrophages) taken from rodents maintained on normal laboratory chow typically contain 15–20% of fatty acids as arachidonic acid and very little very long-chain n-3 fatty acids [18]. The bulk phospholipid of human immune cells (e.g., neutrophils, lymphocytes, monocytes) isolated from subjects of consuming typical Western diets also contains about 20% of arachidonic acid (as a percentage of fatty acids), about 1% EPA and about 2.5% DHA [18]. By modulating lipid raft structure and function and membrane trafficking, we could theoretically reduce the inordinate response of the immune system and lessen the noxious consequences of inflammation [19]. In summary, even though the current human evidence does not allow recommending fish oil supplements, the advice to frequently eat oily fish should be reinforced, albeit the availability of marine products during lockdowns might be lower.

Some people, i.e., 8.7%, decreased fruit and vegetable use and attributed this chance to difficulties in finding open grocery stores in their neighborhood, corroborating the aforementioned findings of Bilal et al. [3] and calling for public health intervention. Alas, half of the participants increased consumption of comfort foods, be them sweet or salty. Whether this is due to the induction of a vicious cycle is still a matter of debate, but there is a clear relation between anxiety levels and cravings/hunger pangs [20]. This agrees with very recent evidence showing that protein consumption is more stable than carbohydrate consumption, suggesting biological control mechanism(s) tightly regulate protein intake and, consequently, influence intake of other macronutrients and food constituents [21]. Conceivably, the adoption of stress-relief techniques such as indoor exercise, meditation, and yoga, might lessen this untoward effect of confinement and should be promoted by health authorities [22].

One issue worth discussing in the 36.8% decrease in alcohol consumption. Alcohol use is a matter of concern by public health authorities; however, it is conceivable that this shift simply reflects the mere substitution of “social drinking”, i.e., alcohol consumption in bars and restaurants with in-house use. In other words, maybe people do not take alcohol use outside of their households into consideration when they compute and report their total consumption. Given the notorious under-reporting of alcohol consumption [23], further investigations should address and elucidate this important issue.

The self-reported decrease in alcohol use was mirrored by an increase in tea, coffee, and herbal tea consumption. Conceivably, the forced creation of a limited space in which to work and live conflated some habits of taking breaks and self-reward. In terms of health effects, tea [24], coffee [25], and herbal teas are very rich in (poly)phenols, namely flavonoids. Plenty of epidemiological studies report inverse associations between (poly)phenol intake and risk of several degenerative diseases, mainly cardiovascular disease, cancer, and neurodegeneration. Intervention trials are not univocal in their conclusions [26], but this result should be view as a positive one, and, hopefully, this habit will be maintained once lockdowns are lifted.

Another interesting result is the decrease in ready-made meals’ purchase, which was halved by the lockdown. Conceivably (and confirmed by anecdotal evidence of yeast and flour scarceness in supermarkets), instant “TV-dinners” are being replaced by hand-made dishes, including homemade bread and cakes. In addition, people have to endure long lines outside of supermarkets, which might have discouraged some to make trips and buy prepared meals, in keeping with the Bilal et al. [3] and Diez et al. [2] data. It should be remembered that part of the healthful effects of the Mediterranean diet is being attributed to the now-disappearing habit [27] of preparing most meals at home and sharing them with friends and family, thereby contributing to a pleasant social environment. We could speculate that if the newfound habit of home cooking is maintained after lockdowns are lifted, future improvement of dietary profiles could be envisaged [28]. As determinants of home cooking are more complex than simply possessing cooking skills, that potential positive associations between cooking, diet, and health require further confirmation [29].

This study has some strengths and limitations that should be acknowledged.

The first strength is that it is the first one of its kind and might provide some guidance to future, larger surveys. Ideally, such surveys should be distributed worldwide as they are easy to implement and yield a great deal of information. This is noteworthy in view of future, inescapable pandemics [30]. Also, we collected a large number of questionnaires, i.e., nearly 2000.

There are also several limitations. One is the young median age of our responder, which is mostly due to the dissemination manner (via social media) and to their familiarity with digital technologies. The latter, also known as the “digital divide” [31], is a frequent bias affecting Internet-based surveys and cannot currently be overcome [32]. Another limitation is the skewing in the geographical distribution of responders, which were 79% from the North-East. This is largely due to the fact that this study was initiated at the University of Padova and, therefore, broadly relied on local participants. Finally, we could not evaluate the impact of lockdown on different population sub-sets, sub-analyzing, e.g., by age, gender, household size, socio-economic status, or ethnicity, which would be important to better target future public health initiatives.

In conclusion, we report the first evidence of dietary habits’ modifications during Covid-19 and associated lockdown. Hopefully, future large-scale similar studies will be undertaken worldwide and will help public health authorities shape their reactions to future, unavoidable pandemics.

## Supplementary Materials

The following are available online at https://www.mdpi.com/2304-8158/9/5/675/s1, Figure S1: Results of the survey.
