# Supplementary material for: Covid-19 and the Subsequent Lockdown Modified Dietary Habits of Almost Half the Population in an Italian Sample
Source: Foods. 2020 May 25;9(5):675. doi: 10.3390/foods9050675 (PMC7278864; doi:10.3390/foods9050675)
Supplement: Supplementary file 1 [file foods-09-00675-s001.pdf]

Sex

1,929 responses

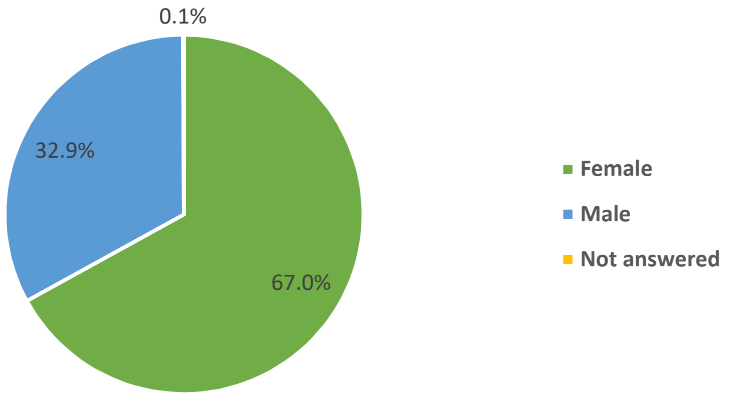

Level of education

1,929 responses

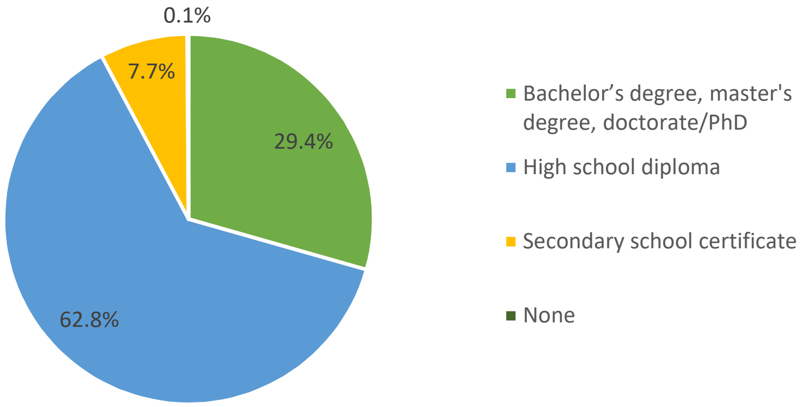

How many people live in your house?

1,929 responses

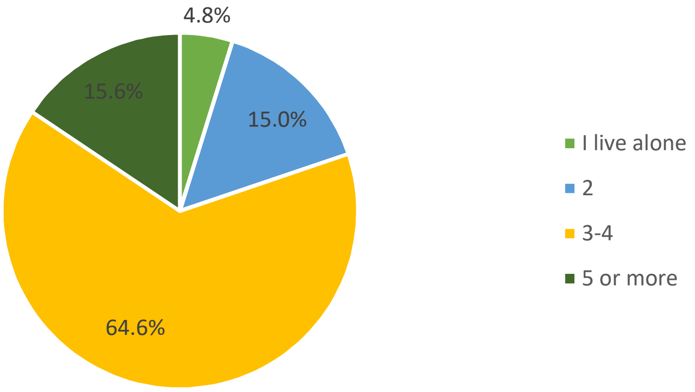

Do you have dependent children?

1,929 responses

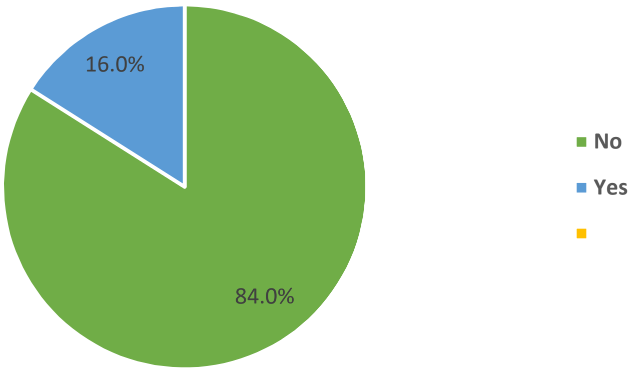

What part of the country do you live in?

1,929 responses

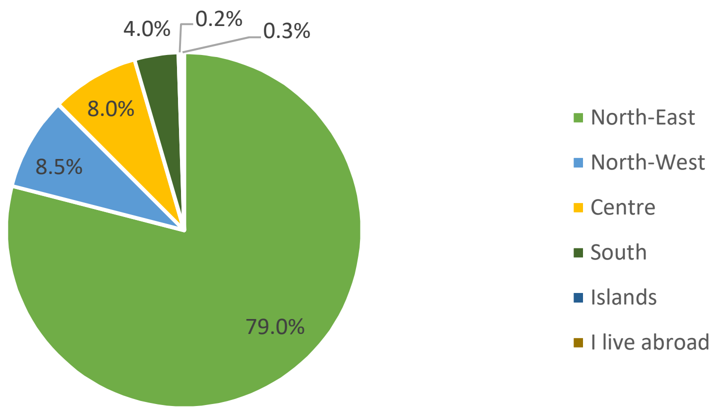

How many portions of fruit and vegetables (fresh or canned) do you usually consume per day?

1,929 responses

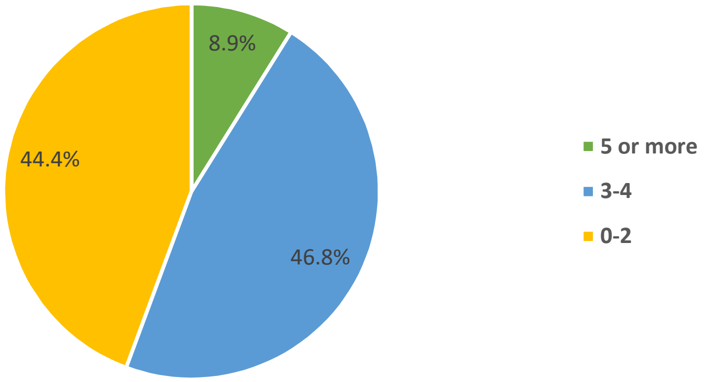

Have you changed your fresh fruit and vegetables consumption during the quarantine?

1,929 responses

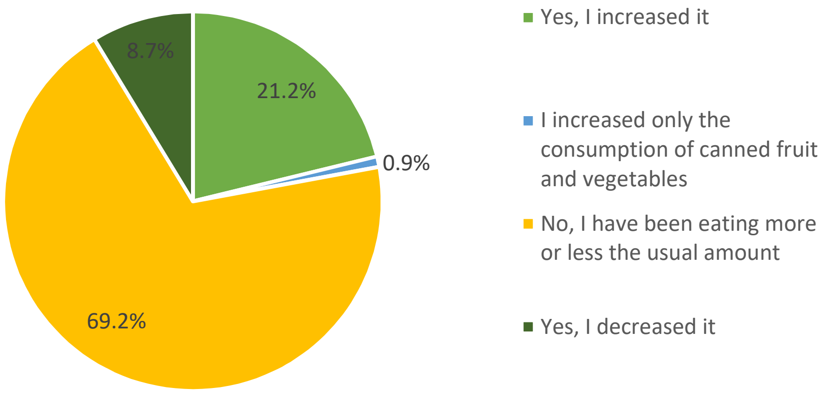

Why have you decreased it?

185 responses

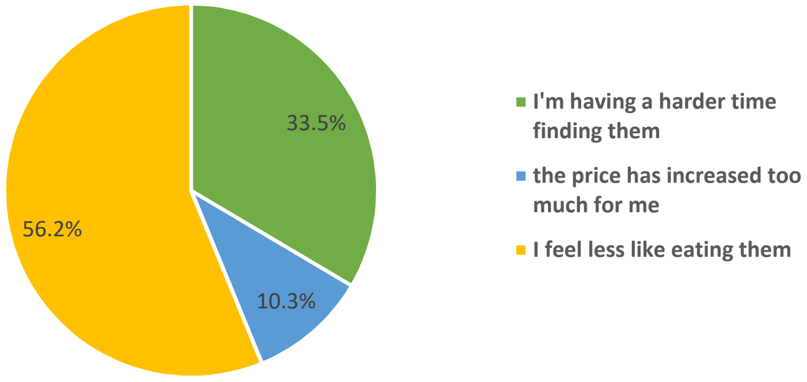

How many times a week do you usually eat red meat?

1,929 responses

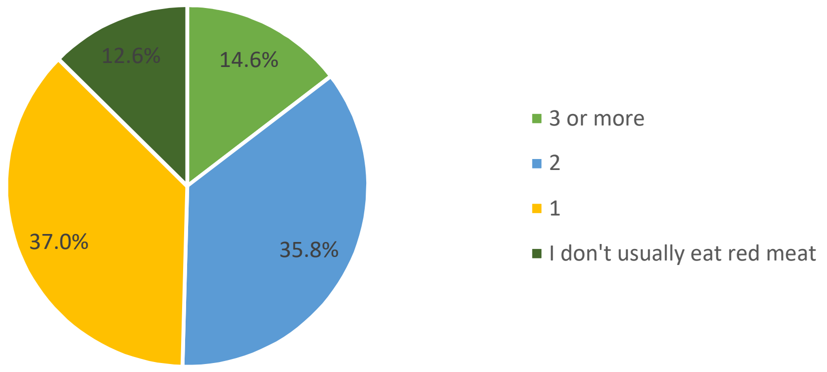

How many times a week do you usually eat processed meat?

1,929 responses

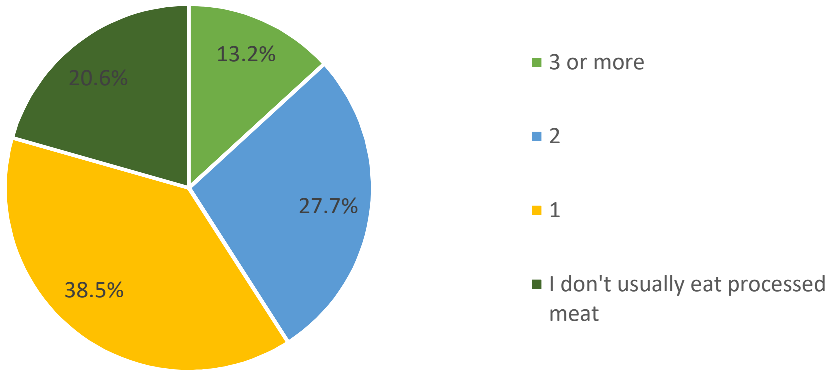

Have you changed your red meat and processed meat consumption during the quarantine?

1,929 responses

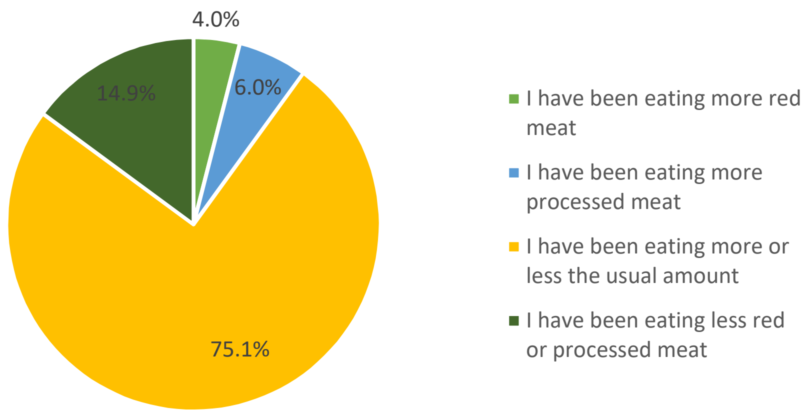

How many portions of fish (fresh or frozen) do you usually consume per week?

1,929 responses

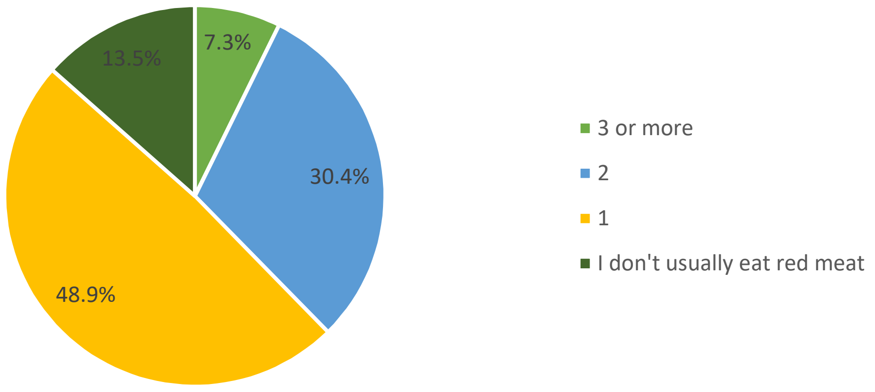

How many portions of canned fish do you usually consume per week?

1,929 responses

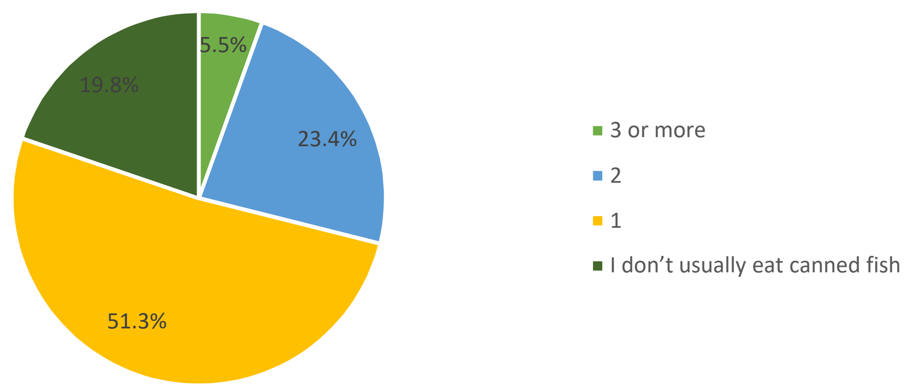

Have you changed your fresh or canned fish consumption during the quarantine?

1,929 responses

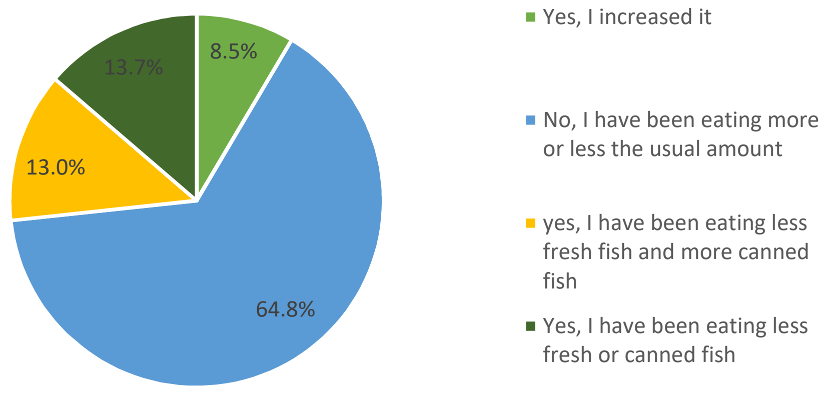

How many glasses of milk/yogurt cups do you usually consume per day?

1,929 responses

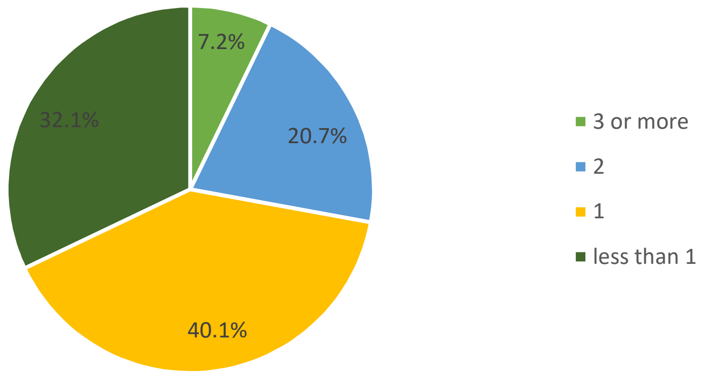

Have you changed your milk/yogurt consumption during the quarantine?

1,929 responses

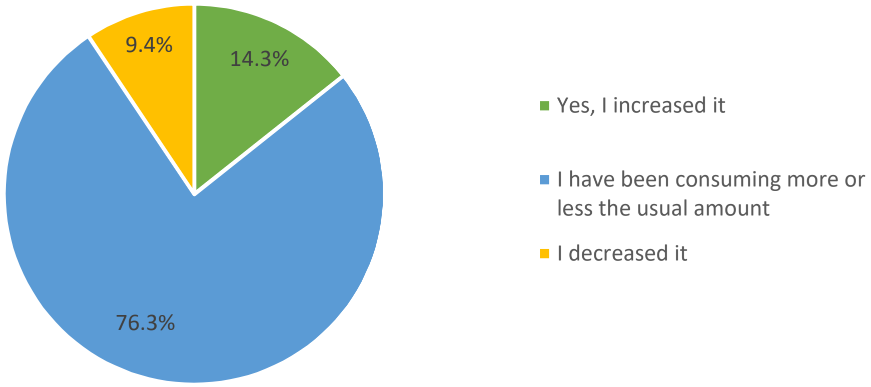

How many portions of cheese (fresh or aged) do you usually consume per week?

1,929 responses

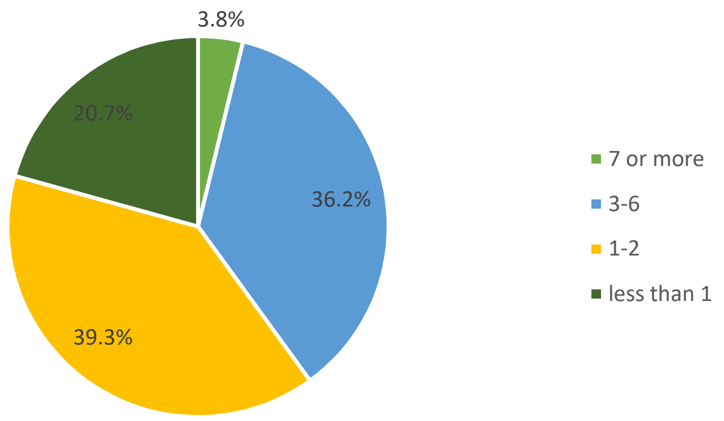

Have you changed your cheese consumption during the quarantine?

1,929 responses

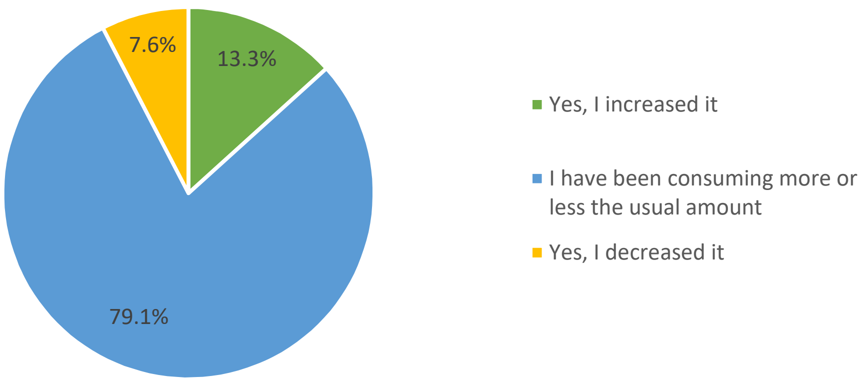

How often do you usually consume biscuits?

1,929 responses

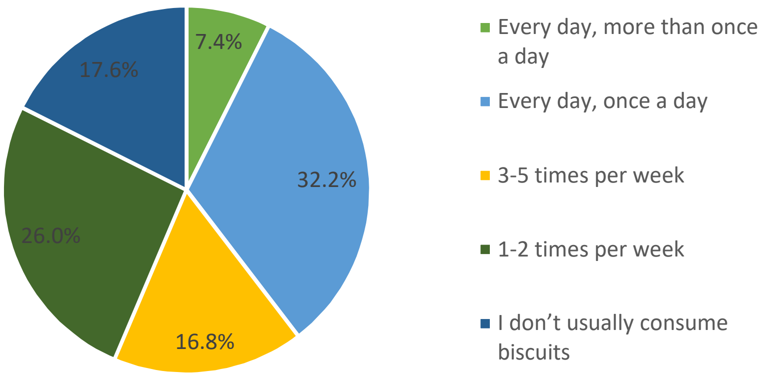

Have you changed your biscuits consumption during the quarantine?

1,929 responses

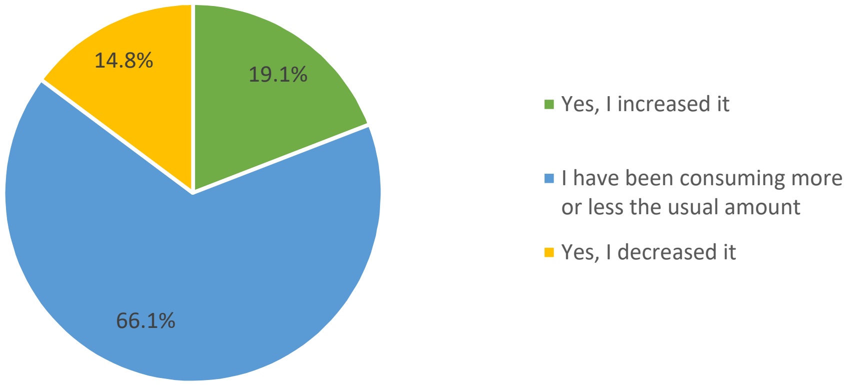

Have you changed your consumption of other kinds of sweet food (chocolate, spreads, cakes, ice creams) during the quarantine?
1,929 responses

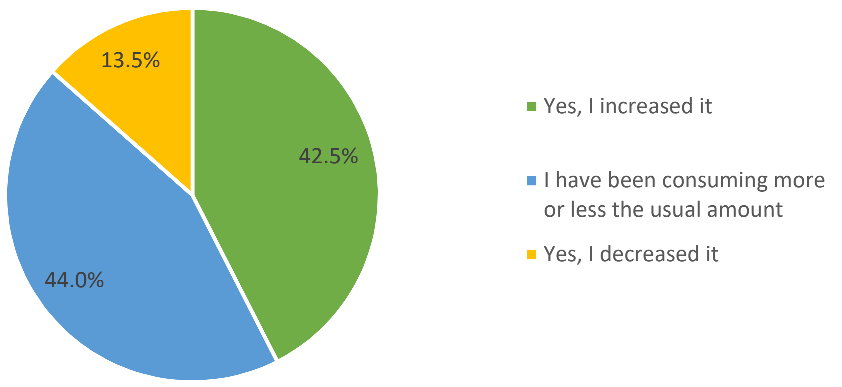

Do you usually use olive oil to cook?
1,929 responses

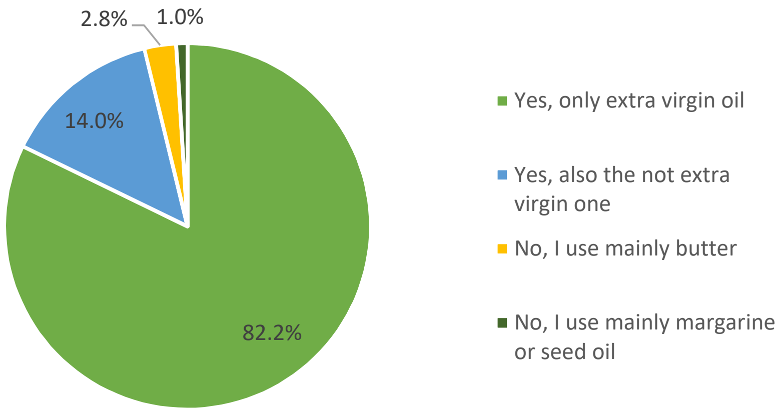

How much olive oil per day do you usually use to season and cook?

1,929 responses

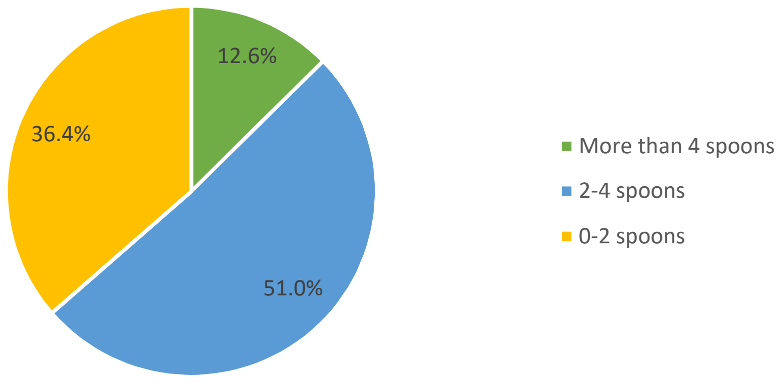

Have you changed your consumption of olive oil during the quarantine?

1,929 responses

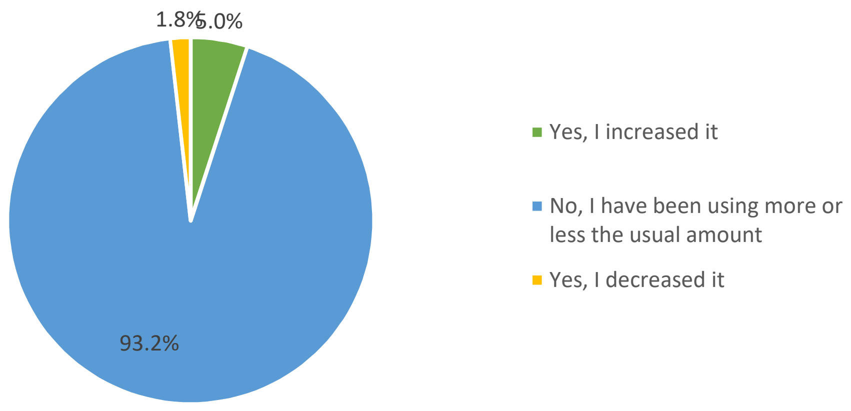

How many cans of sugary drinks (coke, orangeade, tonic, etc.) do you usually consume per week?

1,929 responses

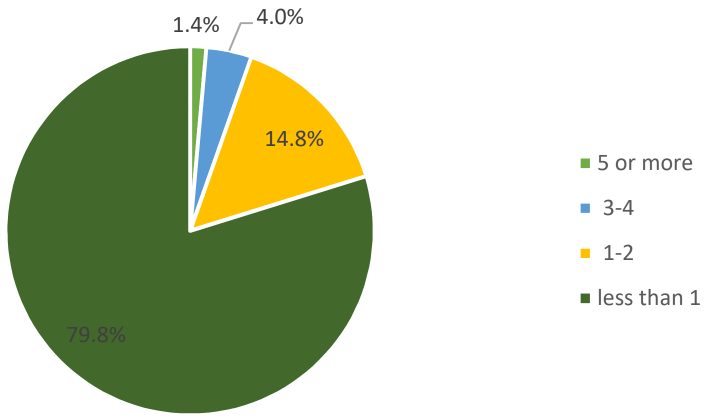

Have you changed your consumption of sugary drinks during the quarantine?

1,929 responses

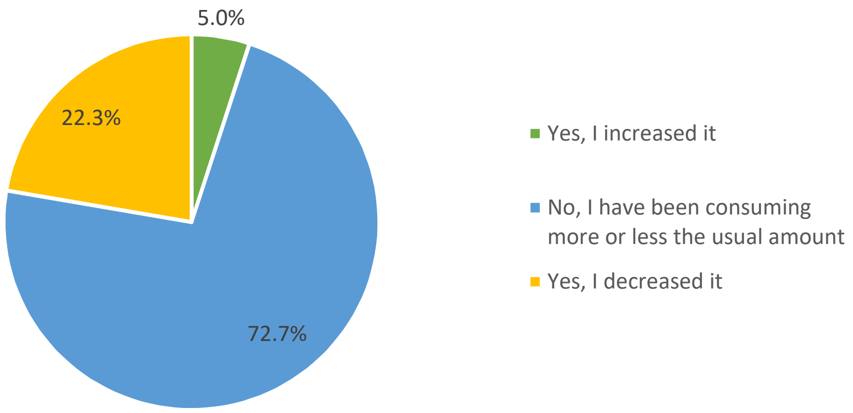

Do you usually drink wine, beer, liqueurs? How many times a day (considering 1 glass of wine/1 can of beer/1 shot of liqueur as 1 standard dose)?

1,929 responses

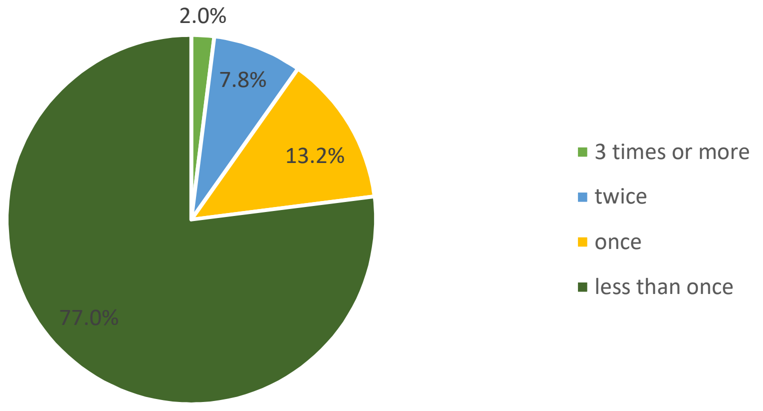

Have you changed your consumption of wine, beer, liqueurs during the quarantine?

1,929 responses

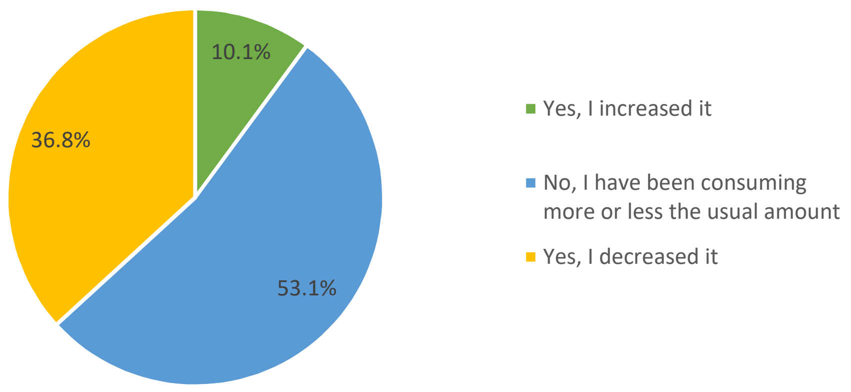

Have you changed your consumption of coffee, tea, infusions during the quarantine?

1,929 responses

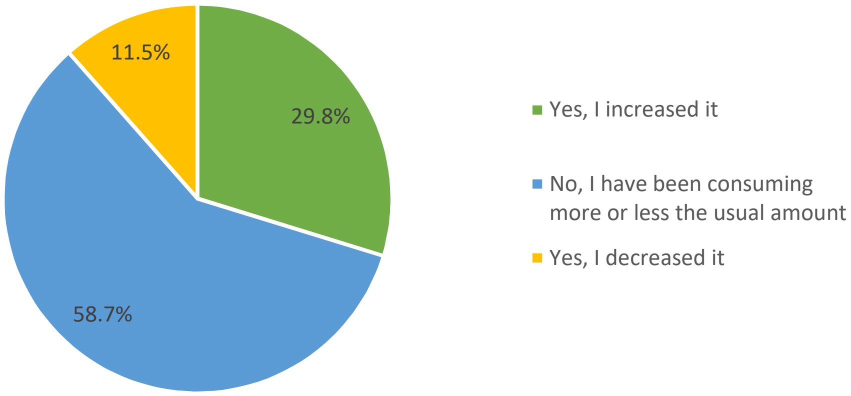

Have you changed your consumption of sweet or salty snacks during the quarantine?

1,929 responses

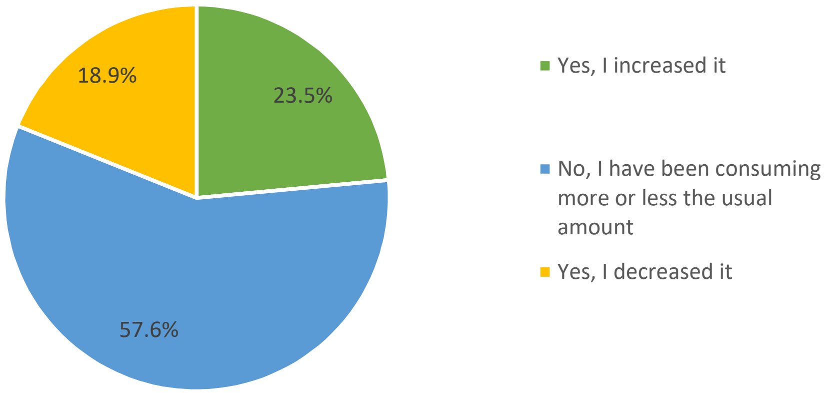

Have you changed your consumption of ready meals during the quarantine?

1,929 responses

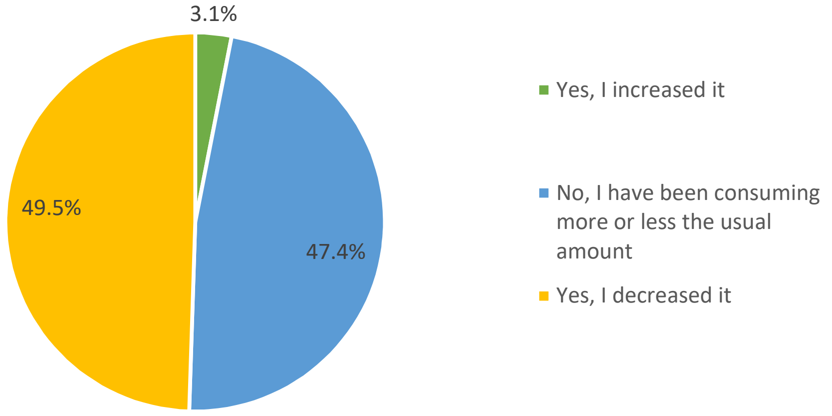

Would you say that you are eating more in this quarantine period?

1,929 responses

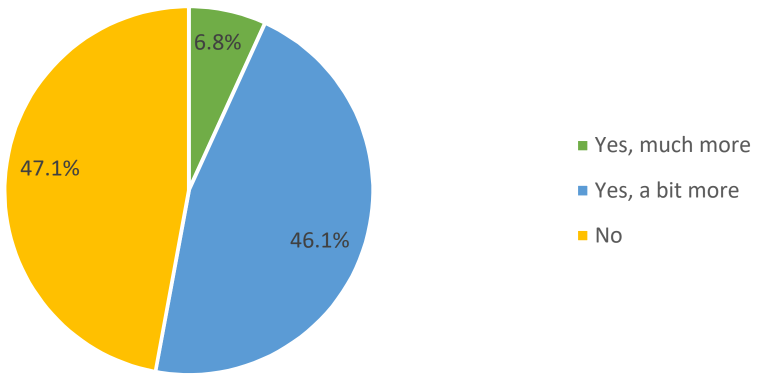

Have you gained weight during the quarantine?

1,929 responses

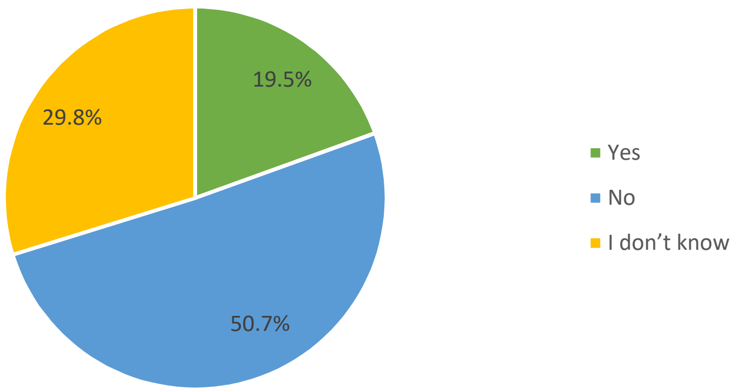

What is the main reason for your diet variations during the quarantine?

1,929 responses

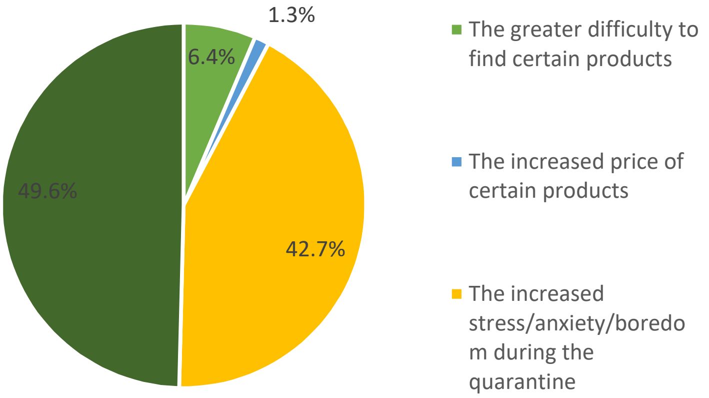

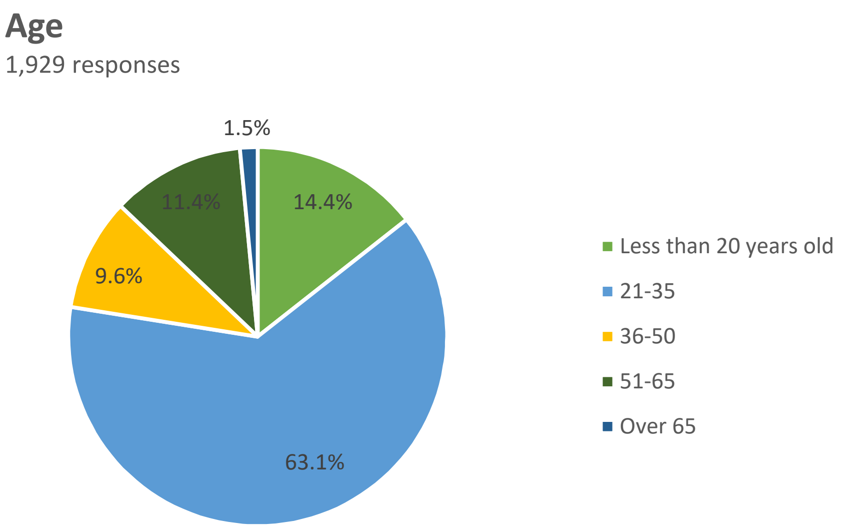

Figure S1 Results of the survey
